# Supplementary material for: “It left me burnt”: Traditional treatment and stigma experiences of cutaneous leishmaniasis in Kalu district, Ethiopia
Source: PLoS Negl Trop Dis. 2026 Feb 23;20(2):e0013537. doi: 10.1371/journal.pntd.0013537 (PMC12948309; doi:10.1371/journal.pntd.0013537)
Supplement: Attachment — Submitted filename: Response to Reviewers.docx [file pntd.0013537.s003.docx]

Date: 24^th^ November 2025

To the Editors

The Journal of PLOS Neglected Tropical Diseases

Re: **PNTD-D-25-01594**

Title: **“It left me burnt”: traditional treatment and stigma experiences of cutaneous leishmaniasis in Kalu district, Ethiopia**

Dear editors and reviewers,

We sincerely appreciate the esteemed reviewers for the valuable and constructive comments. The comments are all considered to fine tune our submission. Enclosed you will find:

- The point-by-point response to your comments.
- The tracked change version of the manuscript showing the appropriate edits
- The clean version of the manuscript

We look forward to receive guidance on the next steps.

Sincerely

Teklu Cherkose, on behalf of the authors

**Point-by-point responses for reviewers’ comments**

Below you will find details of point-by-point response to the comments provided.

**Methods**

**Reviewer #1**: The study provide a clear articulated and testable hypothesis, with a sound study design, however, there are several issues in the methods that need to be addressed. Specifically, the study has a small sample size and unclear selection mechanism reduce confidence that the sample represents the broader CL-infected population in Kalu. Further, the authors only recruited one confirmed CL participant, limiting the representativeness and subgroup interpretation. Additionally the study lacks a comparison group, thus, it is not possible to determine whether reported stigma and experiences are specific to CL rather than common to people with visible facial lesions or skin disease more broadly in the Kalu district. Finally the study only used a single coder thus lacking the ability to measure intercoder agreement or reliability. Thus, the authors cannot ensure coding consistency and credibility in qualitative research, and increase study bias. The authors must: fully describe recruitment, inclusion/exclusion criteria, and nonparticipant data. Additionally, they need include a comparator group and add additional coders to improve the credibility of the results.

**Comment 1: Small sample size and unclear selection mechanism reduce representativeness**

**Response:**

Appreciate this useful observation regarding sample size and representativeness. Our study adopts phenomenological design to document the lived experiences of people affected by CL. Data saturation was ensured with participants representing different profiles with the 18 participants who participated in this study. We have now revised the methods section to more explicitly describe the recruitment process, inclusion criteria, and nonparticipant data.

**Comment 2: Only one confirmed case limits representativeness and subgroup interpretation.**

**Response:**

We appreciate this concern. The objective of this study was to study the lived experiences of affected-people with symptoms of people locally recognized to have CL regardless of formal diagnostic confirmation. This is consistent with available evidence on neglected tropical diseases, where community-recognized illnesses shape social and psychological outcomes. Nonetheless, we have added text in the methods (page 7) acknowledging the diagnostic context in Kalu district, including limited access to laboratory confirmation and clarified that our interest lies in how individual’s experience is shaped by community perceptions, local treatment practices, and access to health services related to CL.

**Comment 3: Lack of a comparison group**

**Response:**

Again, this is useful observation and we appreciate it. As explained in the response above, the objective of this study is to understand the lived experience of those who are believed to have CL in the community. Stigma and psychological experiences were analyzed within the contextual meaning-making processes of participants. The question of whether similar experiences occur among people with other facial or dermatologic conditions is important but beyond the scope of the present study. We have clarified this in the revised manuscript under the limitations section (page 19), noting explicitly that this study does not aim to compare CL stigma with other conditions.

**Comment 4: Only one coder and no intercoder reliability**

**Response:**

Dear reviewer, we acknowledge this point and agree that transparency in qualitative analytic procedures is essential. In phenomenological qualitative analysis, intercoder reliability is not always required nor appropriate because coding relies heavily on the coder’s interpretive engagement with participants’ narratives. Instead, rigor is established through reflexive thematic analysis, peer debriefing, iterative coding and memo writing. We have now strengthened the methodological description (pages 7-9) by:

- Explaining that a reflexive thematic analysis approach was used, in which intercoder reliability is not an indicator of quality.
- Adding details on the reflexive steps undertaken by the primary analyst, including peer debriefing sessions held with senior qualitative researchers on the team to enhance analytic credibility.
- Clarifying how coding decisions were documented and refined through iterative review.

**Reviewer’s** **Suggestions: Authors must include a comparator group and add additional coders**

**Response:**

Dear reviewer, we appreciate the recommendation but respectfully note that incorporating a comparator group or additional coders would fundamentally change the study design and epistemological orientation. The purpose of this qualitative phenomenological study is not to compare illness groups or quantify inter-rater agreement, but to capture the depth and complexity of individual experiences.

**Comment from Reviewer #2:** The objective of the study, to gain information/evidence (rather than data I would argue) to support intervention design is clearly stated. The study population is clearly identified and described. No power calculations were done as this very much a descriptive study of patient experience. No ethical or regulatory concerns.

**Response:**

Thank you for the positive and supportive feedback. We appreciate the recognition that the study’s objective to generate evidence to inform intervention design is clearly articulated. We also welcome the acknowledgment that power calculations are not required for this type of exploratory research and that there are no ethical or regulatory concerns. We have retained and clarified these methodological justifications in the revised manuscript.

**Results**

**Reviewer #1**: The authors provide clear themes and some breakdown of the number of participants that responded within each theme, however, a demographic table of participants is needed. Additionally, please provide a flow chart of potential study participants and why/if they were excluded from the study. Finally, provide a table of all identified themes and how many participants responses fit into each theme.

**Response:**

Thank you for these helpful suggestions aimed at strengthening the clarity and transparency of the results.

**Demographic table:**

We agree that providing participant characteristics enhances reader understanding. We have now added a demographic table summarizing age, gender, lesion location, and lesion status (Table 1) (page 10).

**Flow chart of participant recruitment:**

Dear reviewer, thank you for suggesting us to try to visualize the recruitment process. However, we find it difficult to apply a CONSORT-style or similar flow chart to qualitative research, as recruitment is purposive and iterative rather than sequential or eligibility-based screening. Thus, we improved the narrative explanation of our recruitment process in the revised manuscript to improve clarity and transparency.

**Table of themes with number of contributing participants:**

We acknowledge the value of indicating how broadly each theme was represented across the narratives. We have therefore added a table listing all final themes and subthemes along with the number of participants who contributed to each (Table 2) (page 17).

**Reviewer #2:** Data analysis not applicable.
Descriptive results - I don't think there is any other way to present the report.
No figures, tables etc

**Response to Reviewer #2**

We appreciate the reviewer’s positive assessment of the descriptive presentation of our findings. While the results are indeed narrative in nature, we would like to clarify that we followed established qualitative data analysis procedures, including the use of MAXQDA for data management and an applied thematic analysis approach to systematically develop and refine the narratives presented.

**Conclusions**

**Reviewer #1:** The small sample size, lack of transparency surrounding participant selection, and single coder. limit the accuracy and generalizability of the study. thus, while the manuscript discusses the themes observed in the very small interview sample are as meaningful, general, or causal, they may actually reflect random variation. While some limitations are discussed further discussion surround how these limitations were overcome is needed, accounted for or will be accounted for is needed. Additionally, the limitations listed above need to be addressed.

**Response to Reviewer #1**

Dear reviewer, thank you for these points. As noted in our earlier responses, we have expanded the methods to improve transparency around participant selection, clarified the qualitative rationale for our sample size, and added detail on the analytic procedures used to enhance rigor despite having a single primary coder. We have also updated the limitations section in the discussion to acknowledge these issues and describe how they were addressed within the qualitative framework.

**Reviewer #2:** Study limitations fully acknowledged.
The results are descriptive/narrative but are contextualised into stigma and skin disease milieu. There is no data as such and the number of participants seems on the low side but that is not to dismiss the qualitative information gathered.

**Response to Reviewer #2**

Dear reviewer, thank you for the supportive comments. We appreciate the acknowledgment that the study’s limitations are fully addressed and that, despite the small sample size, the qualitative narratives provide valuable contextual insight into stigma and skin disease experiences.

**Editorial and Data Presentation Modifications?**

Use this section for editorial suggestions as well as relatively minor modifications of existing data that would enhance clarity. If the only modifications needed are minor and/or editorial, you may wish to recommend “Minor Revision” or “Accept”.

**Reviewer #1:**

In line 76, instead of ‘is a skin neglected tropical disease (NTD)’, use ‘is a neglected tropical disease (NTD) of the skin’

Thank you for the suggestion. We rephrased accordingly (see line 76).

In line 77, add a comma after ‘CL is characterized by ulcers, nodules’

Dear reviewer, thank you. We added a comma where requested (see line 78).

In line 87, add a comma after ‘CL affected people experience stigma typified by rejection, isolation’

Dear reviewer, thank you. We added a comma where requested (see line 87).

In line 104, add a comma after ‘in a specific socio-cultural context’

Dear reviewer, thank you. We added a comma where requested (see line 104).

In line 131, the authors state, ‘People with presumed (n=17);’ however, throughout the manuscript, the authors do not make a note or acknowledge this. Rather, they use absolute language such as: ‘All interviewees had CL involving the face’. Please review the manuscript and add language to highlight that while all participants are suspected to have CL, they do not all have confirmed CL. For example, ‘all interviewees had CL-like or CL-presenting symptoms on their faces’.

Dear reviewer, thank you for pointing out this critical comment. We edited the manuscript accordingly.

In line 150, replace which in ‘we present themes which emerged’ with that

Dear reviewer, thank you. We edited as advised (see line 158).

In line 158, replace which in ‘structural stigma which relates’ with that

Dear reviewer, thank you. We edited as advised (see line 166).

In line 351, add a comma after ‘fearing disease progression and death from CL’

Dear reviewer, thank you. We added a comma where requested (see line 365).

In line 355, add the after ‘fear that it would reduce’

Dear reviewer, thank you. We edited as advised (see line 369).

In line 360, add a comma after ‘we recruited had presumed CL’

Dear reviewer, thank you. We added a comma where requested (see line 374).

**Reviewer #2:** Accept. Think this is could be a short letter or article as it is descriptive/qualitative. There is no data to speak of.

**Response:**

We appreciate the reviewer’s positive assessment and their supportive recognition that, although qualitative and descriptive, the manuscript offers meaningful contextual insights.

**Summary and General Comments**

**Reviewer #1:** The manuscript ‘“It left me burnt”: traditional treatment and stigma experiences of cutaneous leishmaniasis in Kalu district, Ethiopia,’ by Cherkose et al., offers novel qualitative insight into the physical, emotional, and social effects of cutaneous leishmaniasis (CL) in residents of Kalu district, Ethiopia. Few studies have examined these factors and fewer still have looked at a Kalu district, Ethiopia specifically. Additionally, the examination of traditional medicine and the avoidance behavior is a novel addition. However, serious methodological shortcomings undermine confidence in the findings. The small sample size with poorly described recruitment methods introduces clear selection bias and limited generalizability. Further, the absence of any counterfactual or comparator group prevents the determination of whether reported practices and suffering are specific to CL. Finally, the manuscript lacks a transparent thematic analysis description and only uses a single coder, allowing selective reporting and a lack of intercoder reliability.

**Revision:** fully describe recruitment, inclusion/exclusion criteria, and nonparticipant data. Additionally, they need to report diagnostic status for each participant and either include a comparator group or reframe claims as exploratory and non-specific to CL. Additionally, the authors must present a reproducible analytic approach with a codebook, counts for each theme in a table format, and perform inter-coder procedures with the addition of a second coder and a tie breaker coder for disagreements.

**Response:**

Dear reviewer, we appreciate these suggestions; however, because our study is a phenomenological qualitative inquiry rather than a comparative or quantitative design, requirements such as a comparator group, diagnostic reporting tables, codebooks, theme counts, and multi-coder reliability procedures are not applicable, and the study has been appropriately framed as exploratory with recruitment details and analytic procedures already transparently described in the text.

**Reviewer #2:** I think this is a neat paper. I am familiar with CL as an infectious disease but have less knowledge about the stigma associated with skin NTDs and how that stigma manifests. My concerns are around the sample size and lack of parasitological confirmation but I do think the results show the importance of improved awareness around CL for those affected and for the communities involved. It is a well written and presented manuscript clearly identifying the need to understand the existing context of CL in an area prior to designing a putative intervention.

**Response:**

Dear reviewer, thank you for the encouraging feedback and thoughtful reflections. We acknowledge the concerns regarding sample size and limited parasitological confirmation. As noted in our revisions, we have clarified the qualitative rationale and contextual diagnostic challenges. We appreciate the recognition that the findings underscore the importance of understanding local experiences and stigma surrounding CL to inform context-appropriate interventions.

**Summary of revisions made**

- Expanded description of recruitment, inclusion criteria, and nonparticipant data.
- Clarified the diagnostic context and rationale for including community-recognized cases.
- Strengthened description of analytic rigor, including reflexivity and peer debriefing.
- Added limitations reflecting the issues raised.
