# Supplementary material for: “It left me burnt”: Traditional treatment and stigma experiences of cutaneous leishmaniasis in Kalu district, Ethiopia
Source: PLoS Negl Trop Dis. 2026 Feb 23;20(2):e0013537. doi: 10.1371/journal.pntd.0013537 (PMC12948309; doi:10.1371/journal.pntd.0013537)
Supplement: Attachment — Submitted filename: Manuscript review Cherkose et al. contd.docx [file pntd.0013537.s002.docx]

Review for: “It left me burnt”: traditional treatment 2 and stigma experiences of cutaneous 3 leishmaniasis in Kalu district, Ethiopia”

The revised manuscript ‘“It left me burnt”: traditional treatment and stigma experiences of cutaneous leishmaniasis in Kalu district, Ethiopia,’ by Cherkose et al., offers novel qualitative insight into the physical, emotional, and social effects of cutaneous leishmaniasis (CL) in residents of Kalu district, Ethiopia. The revised manuscript appropriately addresses or acknowledges several significant methodological shortcomings that previously undermined confidence in the findings. The authors have now recognized the limitation related to presumed versus confirmed VL, as well as described the recruitment methodology, thematic interpretation process, and the absence of a counterfactual. However, several additional methodological issues remain unresolved. Specifically, these include inconsistencies within the demographic table, limited detail on the approach used to confirm agreement, and potential biases introduced by the combination of purposive sampling and snowball referrals. Therefore, while the authors have made progress in addressing several key issues, the manuscript requires additional substantive revisions before it can be considered for acceptance.

**Major Comments**

1. Demographic Table Error

In the manuscript, the authors state that "eleven of the 18 people interviewed were male"; however, the demographic table lists 13 males. This discrepancy should be corrected. Furthermore, the table should not include patient identification numbers. Instead, it should be structured with balanced variables that stratify participant groups, allowing readers and reviewers to accurately interpret the data distribution. Rather than listing each participant, the authors should present pooled counts stratified by the selected variables.

2. Method for Confirming Agreement

The manuscript mentions that agreement was confirmed, but provides little information on how this was achieved. The authors should briefly describe the method used to confirm agreement, including whether multiple analysts were involved and if a tie-breaking process was implemented. This information is essential to support the credibility and validity of the identified themes in the results.

3. Sampling Method Limitations

Although purposive sampling is an appropriate qualitative approach, it introduces potential selection bias that restricts generalizability. Because participants are chosen based on the interviewer's discretion, the resulting sample may not adequately represent broader populations. The additional use of snowball sampling could further compound this effect, as participants are likely to refer others with similar characteristics, thereby increasing homogeneity. These limitations must be clearly acknowledged and discussed within the manuscript.
